# Draft Genome Sequences of Two Vibrio fortis Strains Isolated from Coral (*Fungia* sp.) from the Andaman Sea

**DOI:** 10.1128/MRA.01225-19

**Published:** 2020-01-02

**Authors:** Sushanta Deb, Jhasketan Badhai, Subrata K. Das

**Affiliations:** a Department of Biotechnology, Institute of Life Sciences, Bhubaneswar, India; Loyola University Chicago

## Abstract

We report the draft genome sequences of Vibrio fortis strains AN-60 and S7-72, which were isolated from coral (*Fungia* sp.) from the Andaman Sea. The genome sizes for strains AN-60 and S7-72 are 5.43 and 5.53 Mb, respectively. Both strains harbor genes associated with protocatechuate and azathioprine degradation and the sulfate reduction pathway.

## ANNOUNCEMENT

The genus *Vibrio* consists of a large number of species, including species commensal and pathogenic to several marine organisms (1). Vibrio fortis is a facultatively anaerobic, nonswarming, slightly curved bacterium. This bacterium was first isolated from aquatic animals and the marine environment (2). Studies have reported Vibrio fortis strains causing disease in seahorses and sea urchins (3, 4). To date, the whole-genome sequence of only one Vibrio fortis strain has been reported (4). As a result, limited information is available for understanding the evolution, ecology, and pathogenicity of this bacterium.

Bacterial strains used in this study were isolated from corals from the Andaman Sea. The collection of coral samples, isolation of bacteria, and growth conditions were described earlier (5). Genomic DNA was isolated using the QIAamp DNA minikit (Qiagen, Germany). The quality (*A*_260_/*A*_280_ ratio) and concentration of DNA were determined using a NanoDrop 8000 UV-visible spectrophotometer and a Qubit 2.0 fluorometer (Thermo Fisher Scientific, USA), respectively. DNA was sheared to an average length of 10 kb using a g-TUBE device, according to the manufacturer’s protocol (Covaris, Woburn, MA, USA). Fragmented DNA was used for SMRTbell library preparation, as recommended by the manufacturer. The quantity and quality of the SMRTbell libraries were evaluated using a high-sensitivity double-stranded DNA kit with the Qubit fluorometer and a DNA 12000 kit with a 2100 bioanalyzer (Agilent, Santa Clara, CA, USA), respectively. Sequencing was performed with the PacBio Sequel sequencing system (Pacific Biosciences, USA).

*De novo* genome assembly of PacBio reads was performed with the Canu 1.3 assembler (https://github.com/marbl/canu) (parameters were as follows: correct; p, bacteria; merylMemory, 15; batThreads, 12; stopOnLowCoverage , 100; genomeSize, 5.2m) (6). The scaffolding was performed using the Single Molecular Integrative Scaffolding (SMIS) pipeline (https://github.com/fg6/smis) (parameters were as follows: score, 50; len, 2,000; step, 200; contig, 3,000; edge, 5). Finally, the gaps were filled using PBJelly (parameters were as follows: minMatch, 8; minPctIdentity, 70; bestn, 1; nCandidates, 10; maxScore, 500; nproc, 8; noSplitSubreads) (7). A Perl script (https://github.com/tomdeman-bio/Sequence-scripts/blob/master/calc_N50_GC_genomesize.pl) was used to calculate the statistical elements of the assembled genome (Table 1). The draft genomes were annotated using the NCBI Prokaryotic Genome Annotation Pipeline (PGAP version 4.9) (8). The final draft genome assemblies of strains AN-60 and S7-72 are summarized in Table 1. Putative pathways in the bacterial genomes were identified by using a KEGG pathway analysis tool (9). The Clusters of Orthologous Groups (COG) functional categories of the predicted protein-coding genes were identified using the Perl script cdd2cog (https://github.com/aleimba/bac-genomics-scripts/tree/master/cdd2cog) (10).

**TABLE 1:** Characteristics and accession numbers of the draft genomes of Vibrio fortis strains AN60 and S7-72

Strain	GenBank accession no.	Genome size (Mb)	GC content (mol%)	No. of scaffolds	No. of reads	*N*_50_ (Mb)	No. of coding sequences	% of genes encoding proteins	No. of tRNAs	No. of rRNAs
AN60	VWSE00000000	5.43	45.06	11	460,416	1.19	4,755	94.55	137	44
S7-72	VXDD00000000	5.53	44.94	7	1,258,001	2.83	4,873	95.81	134	47

For identification of the strains, 16S rRNA gene sequence analysis was performed, and the results were compared with the EzBioCloud database (11). Strain AN-60 showed 99.86% and strain S7-72 showed 99.73% 16S rRNA gene sequence similarity to Vibrio fortis LMG21557^T^. In addition, the average nucleotide identity (ANI) with reference Vibrio fortis strain Dalian14 was determined using the JSpeciesWS server (12). The ANIs of strains AN-60 and S7-72 with the reference strain were above the threshold value of 96%, indicating that the strains represented the same species of Vibrio fortis. COG functional analysis revealed that the genomes of strains AN60 and S7-72 contained genes involved in coenzyme transport and metabolism (1.34% and 3.40%, respectively), ribosomal structure and biogenesis (3.99% and 4.18%, respectively), replication, recombination, and repair (3.48% and 4.95%, respectively), and cell motility (2.59% and 3.26%, respectively). Further, putative genes or gene clusters for protocatechuate and azathioprine degradation and sulfate reduction pathways were found in the genomes. Thus, the genomic information for strains AN-60 and S7-72 might facilitate an understanding of the degradation of various aromatic and toxic compounds by marine *Vibrio* spp.

### Data availability.

The whole-genome shotgun sequences of strains AN60 and S7-72 have been deposited in DDBJ/ENA/GenBank under accession numbers VWSE00000000 and VXDD00000000, respectively. SRA data are available in the NCBI SRA database under accession numbers SRR10194532 and SRR10194576, respectively.

## References

[1] Romalde JL, Diéguez AL, Lasa A, Balboa S 2014 New *Vibrio* species associated to molluscan microbiota: a review. Front Microbiol 4:413. doi:10.3389/fmicb.2013.00413. 24427157 PMC3877837

[2] Thompson FL, Thompson CC, Hoste B, Vandemeulebroecke K, Gullian M, Swings J 2003 *Vibrio fortis* sp. nov. and *Vibrio hepatarius* sp. nov., isolated from aquatic animals and the marine environment. Int J Syst Evol Microbiol 53:1495–1501. doi:10.1099/ijs.0.02658-0. 13130038

[3] Wang X, Zhang Y, Qin G, Luo W, Lin Q 2016 A novel pathogenic bacterium (*Vibrio fortis*) causing enteritis in cultured seahorses, *Hippocampus erectus* Perry, 1810. J Fish Dis 39:765–769. doi:10.1111/jfd.12411. 26466548

[4] Ding J, Dou Y, Wang Y, Chang Y 2014 Draft genome sequence of *Vibrio fortis* Dalian14 isolated from diseased sea urchin (*Strongylocentrotus intermedius*). Genome Announc 2:e00409-14. doi:10.1128/genomea.00409-14. 24994792 PMC4081992

[5] Badhai J, Kumari P, Krishnan P, Ramamurthy T, Das SK 2013 Presence of SXT integrating conjugative element in marine bacteria isolated from the mucus of the coral *Fungia echinata* from Andaman Sea. FEMS Microbiol Lett 338:118–123. doi:10.1111/1574-6968.12033. 23083057

[6] Koren S, Walenz BP, Berlin K, Miller JR, Bergman NH, Phillippy AM 2017 Canu: scalable and accurate long-read assembly via adaptive k-mer weighting and repeat separation. Genome Res 27:722–736. doi:10.1101/gr.215087.116. 28298431 PMC5411767

[7] English AC, Richards S, Han Y, Wang M, Vee V, Qu J, Qin X, Muzny DM, Reid JG, Worley KC, Gibbs RA 2012 Mind the gap: upgrading genomes with Pacific Biosciences RS long-read sequencing technology. PLoS One 7:e47768. doi:10.1371/journal.pone.0047768. 23185243 PMC3504050

[8] Tatusova T, DiCuccio M, Badretdin A, Chetvernin V, Nawrocki EP, Zaslavsky L, Lomsadze A, Pruitt KD, Borodovsky M, Ostell J 2016 NCBI prokaryotic genome annotation pipeline. Nucleic Acids Res 44:6614–6624. doi:10.1093/nar/gkw569. 27342282 PMC5001611

[9] Kanehisa M, Sato Y, Morishima K 2016 BlastKOALA and GhostKOALA: KEGG tools for functional characterization of genome and metagenome sequences. J Mol Biol 428:726–731. doi:10.1016/j.jmb.2015.11.006. 26585406

[10] Leimbach A 2016 Bac-genomics-scripts: bovine *E. coli* mastitis comparative genomics edition Zenodo. doi:10.5281/zenodo.215824.

[11] Kim O-S, Cho Y-J, Lee K, Yoon S-H, Kim M, Na H, Park S-C, Jeon YS, Lee J-H, Yi H, Won S, Chun J 2012 Introducing EzTaxon-e: a prokaryotic 16S rRNA gene sequence database with phylotypes that represent uncultured species. Int J Syst Evol Microbiol 62:716–721. doi:10.1099/ijs.0.038075-0. 22140171

[12] Richter M, Rosselló-Móra R, Glöckner FO, Peplies J 2016 JSpeciesWS: a Web server for prokaryotic species circumscription based on pairwise genome comparison. Bioinformatics 32:929–931. doi:10.1093/bioinformatics/btv681. 26576653 PMC5939971

